# Soil resilience and recovery: rapid community responses to management changes

**DOI:** 10.1007/s11104-016-3068-x

**Published:** 2016-09-30

**Authors:** Penny R. Hirsch, Deveraj Jhurreea, Jennifer K. Williams, Philip J. Murray, Tony Scott, Tom H. Misselbrook, Keith W. T. Goulding, Ian M. Clark

**Affiliations:** 1 Rothamsted Research, Harpenden, Hertfordshire AL5 2JQ UK; 2 Rothamsted Research, North Wyke, Okehampton, Devon EX20 2SB UK

**Keywords:** Soil microbiome, Soil bacteria, Soil fungi, Soil mesofauna, Soil organic carbon, Grass, Wheat, Bare fallow soil, Nitrogen-cycling genes

## Abstract

**Background and aims:**

Soil degradation is a major global problem; to investigate the potential for recovery of soil biota and associated key functions, soils were monitored during the early years of conversion between permanent grassland, arable cropping and bare fallow (maintained by regular tilling). Distinct differences in soil properties had become apparent 50 years after a previous conversion.

**Methods:**

Subplots on previously permanent grassland, arable and bare fallow soil were converted to the two alternatives, generating 9 treatments. Soil properties (soil organic carbon, mesofauna, microbial community structure and activity) were measured.

**Results:**

After 2 years, mesofauna and microbial abundance increased where plants were grown on previously bare fallow soils and declined where grassland was converted to bare fallow treatment. Overall prokaryote community composition remained more similar to the previous treatments of the converted plots than to the new treatments but there were significant changes in the relative abundance of some groups and functional genes. Four years after conversion, SOC in arable and bare fallow soils converted to grassland had increased significantly.

**Conclusions:**

Conversion to permanent grassland effectively replenished C in previously degraded soil; the soil microbiome showed significant conversion-related changes; plant-driven recovery was quicker than C loss in the absence of plants.

**Electronic supplementary material:**

The online version of this article (doi:10.1007/s11104-016-3068-x) contains supplementary material, which is available to authorized users.

## Introduction

The relationship between plants and soil is fundamental to life on earth. Photosynthesis enables plants to supply energy-rich organic carbon compounds to the soil community which in turn makes essential nutrients available. The absence of plants can lead to desertification, with microbial DNA below the limits of detection (Navarro-Gonzalez et al. 2003). In contrast, soil organic matter and soil organic carbon (SOC) are more strongly associated with improved soil quality (Johnston et al. 2009) than reducing tillage which results in only modest increases in SOC in the UK (Powlson et al. 2012). Soils in England and Wales are reported to be losing organic C at a mean rate of 0.6 % per year (Bellamy et al. 2005) and converting land under arable cultivation to grassland has been proposed to improve soil quality, however there are concerns that reversion of such land will lead to increased greenhouse gas emissions and losses of sequestered C (Six 2013). Understanding the processes leading to decline or accumulation of SOC is therefore highly relevant to climate change.

The soil biota plays a crucial role in SOC dynamics, essential for maintaining the soil food web and cycling C and N. They are potentially affected by land use including plant cover, tillage and fertilizer inputs (de Vries et al. 2013). Many studies show differences in communities from soils with contrasting treatments (Fierer et al. 2012; Garbeva et al. 2008; Geisseler and Scow 2014; de Quadros et al. 2012; Zhalnina et al. 2013). In non-agricultural systems there is evidence for the development of distinct bacterial communities selected over centuries (Cutler et al. 2014; Jangid et al. 2011) and fungal communities over decades (Hogberg et al. 2014) with soil pH a major factor in microbial community structure at continental, landscape and field scale (Fierer and Jackson 2006; Griffiths et al. 2011; Zhalnina et al. 2015). Soil factors including pH and C: N ratio had a greater influence than land use on bacterial communities in Netherlands soil (Kuramae et al. 2011), although these factors will also be influenced by agricultural land use. Perennial cover such as grass will provide a constant source of both recalcitrant and labile C to the soil biota compared to annual wheat crops, but the latter also receive fertilizer to increase yield quality and quantity. Increased N supply will favour certain groups of microorganisms including nitrifiers that oxidize ammonia and nitrite as an energy source, and denitrifiers that can use nitrate as an electron acceptor when conditions become anaerobic. Perennial untilled and arable, fertilized, tilled treatments appear to favour distinct groups of microorganisms; different from communities in soil receiving no plant or fertilizer inputs (Zhalnina et al. 2013). In addition to these overarching effects, there are temporal fluctuations in soil microbial community structure within replicate plots, albeit less marked than between treatments (Lauber et al. 2013). Although comparison of long-term treatments provides evidence that soil management influences soil biota (Hirsch et al. 2009; Zhalnina et al. 2013, 2015), there is scant information on the responses during the early years following change.

In the Highfield experiment at Rothamsted Research, UK, where plant growth has been suppressed in bare fallows since 1959, there has been a marked decline in soil C (Coleman et al. 1997), aggregate structure (Watts et al. 2001) and microbial and mesofaunal abundance (Hirsch et al. 2009). This experiment, set up on a site that had been under pasture for centuries, compares the bare fallow treatment in which plants are regularly removed by tilling, plots converted to continuous arable (wheat) cultivation in 1949, or maintained as permanent grassland. An interesting earlier observation was that despite a reduction in bacterial abundance in the bare fallow soil, the number of operational taxonomic units (OTU) detected was similar to that in the grassland soil (Hirsch et al. 2009). A separate study showed reductions in microbial biomass compared to grassland of five-fold in arable and ten-fold in bare fallow plots (Wu et al. 2012). Although marked differences in soil C in the contrasting Highfield plots had become apparent after 50 years, there was no information on short-term changes in SOC, soil microbial and mesofaunal communities, or key genes and functions.

To address the hypothesis that changes in land use have rapid and significant effect on the soil biota, and that replanting can exert rapid recovery in C-starved soil, subplots within each long-term Highfield treatment (grass, arable, bare fallow) were converted to the alternative treatments and soil properties and biota were measured at intervals over the following 4 years.

## Methods

### Conversion

In October 2008, 10 × 6 m areas within the existing bare fallow, arable and grassland plots on the Highfield/Geescroft site of Rothamsted farm were converted to one of the alternative treatments. Plots were subdivided in a randomized block design to provide three plots for each permanent and conversion treatment, resulting in a total of 27 plots and 9 treatments. Each treatment was separated by 10 m with a buffer zone of 1 m at each end and 0.5 m at each side to reduce edge effects, giving a central area of 8 × 5 m for sampling. All plots except those remaining as permanent grassland were ploughed (standard depth, 23 cm) and the first sampling was made a few days later. To minimise the effects of differences in soil pH, prior to conversion the bare fallow soil was adjusted from pH 5.8 to pH 6.5 (by addition of ground chalk) in line with the arable and grassland soils. Arable and grass plots were fertilized to provide 65 kg P, 250 kg K ha^−1^ (repeated every 3 years on arable plots); arable plots were planted with wheat (Hereward, 350 seeds m^−2^, coated with standard Bayer insecticide/fungicide treatment Redigo Deter); conversions to grassland with a fescue/timothy grass/white clover mix (30 kg ha^−1^). Ammonium nitrate fertilizer applied to arable plots in the spring, in three doses, provided approximately 220 kg N ha^−1^y^−1^ in total. The bare fallow plots were maintained with regular tillage, ploughed or rotavated at least four times per year to keep weeds to a minimum. Arable and grassland meadow plots were maintained under standard Rothamsted farm practice with grass mowed twice during the summer; wheat straw and hay removed; wheat straw and grain yields measured at harvest.

### Soil sampling

Due to practical limitations, not all parameters could be measured each year and we used our previous experience (Hirsch et al. 2009; Clark et al. 2012) to plan sampling to cover the most relevant periods of change. A randomised sampling grid was designed at the outset of the conversion to provide 160 sampling areas of 0.25 m^2^ to ensure that no part of each plot was sampled more than once and different assays did not interfere. A timeline of sampling soil for different analyses is in shown in Table 1. At each sampling time, three randomly assigned grid areas were sampled to 10 cm depth using a 3 cm dia. corer with the top 2 cm containing root mats and other plant detritus discarded. Ten cores for each grid area were pooled and thoroughly mixed whilst sieving through 2 mm mesh; samples were then processed immediately (e.g. for dry weight estimations) or frozen at −80 °C for subsequent analyses. All implements were cleaned with 70 % ethanol between sampling/sieving soil from each grid area. This provided three replicates from each plot to give 9 replicates per treatment. DNA was extracted from 250 mg soil samples using the PowerSoil® kit (MoBio Laoratories, Inc. (Carlsbad, CA USA) as described previously (Clark et al. 2012). DNA quality was assessed using a Nanodrop® ND-2000 Spectrophotometer (Thermo Fisher Scientific, Wilmington, DE, USA), quantified using a Qubit® 2.0 Fluorimeter and ds DNA HS assay kit (Thermo Fisher), and presented as yield g^−1^ dry soil.

**Table 1:** Timeline for sampling and analysing the Highfield conversion experiment

Date	Year 1	Year 2	Year 3	Year 4	Year 5
	October 2008	October 2009	October 2010	October 2011	October 2012
State of conversion	Start: creation of conversion plots within permanent treatments by ploughing and/or sowing wheat/grass	Preliminary consequences of perturbation	Changes in soil invertebrates and functional microbial genes	Changes in microbial diversity	Changes in total soil C and fractions
Samples	N_2_O emissions measured fortnightly	Intact cores			
	Soil sampled	Soil sampled	Soil sampled	Soil sampled	Soil sampled
	DNA extracted	DNA extracted	DNA extracted	DNA extracted	
Analyses	Total C & N	Total C & N	Total C & N	Total C & N	Total C & N
					Light detritus
	16S rRNA gene abundance	16S rRNA gene abundance	16S rRNA gene abundance	16S rRNA gene abundance	
			N-cycling gene abundance		
	Amplicon sequencing for bacterial diversity		Invertebrates counted from intact cores	Amplicon sequencing for bacterial & fungal diversity	

### Analyses of phylogenetic and functional genes

DNA extracted from one or two soil samples from each replicate (up to 18 per treatment) was subjected to real-time qPCR analyses to assess abundance of the total bacterial community 16S rRNA genes and functional genes for denitrifying bacteria (*nirK, nirS* and *nosZ*) and the ammonia monooxygenase gene *amoA* from nitrifying bacteria and archaea. DNA samples and PCR results failing to meet our quality criteria were discarded: a full description of the primers, PCR conditions and quality controls has been described previously (Cardenas et al. 2013; Clark et al. 2012) and is provided in the Supplementary Information. We did not quantify archaeal 16S rRNA genes: previous results indicated that they were 100-times less frequent than bacteria in our soils and the vast majority were ammonia oxidizers, so would be enumerated by the primers for archaeal *amoA* (Zhalnina et al. 2013).

For community analysis using amplicon sequencing, one pooled DNA sample from each plot was used, providing 3 replicates per treatment. Both bacterial and archaeal 16S rRNA genes were amplified using barcoded universal prokaryotic primers 515F/806R (Caporaso et al. 2011), sequenced using Illumina’s MiSeq platform (NGS Core Facility, Argonne National Laboratory, Leemont, IL, USA) and analysed using the QIIME 1.8 pipeline (Caporaso et al. 2010, 2012). Fungal ITS genes were amplified using barcoded universal primers ITS 1F (CTTGGTCATTTAGAGGAAGTAA, Gardes and Bruns 1993) and ITS4 (TCCTCCGCTTATTGATATGC, White et al. 1990), sequenced on a Roche 454 GS-FLX and fungal ITS amplicon data analyzed using QIIME 1.8 /UNITE (Abarenkov et al. 2010), reference data sets from November 2012 incorporating UCHIME reference dataset (2014-07-26, 21059 sequences) for chimera removal (Edgar et al. 2011). Bacterial and fungal operational taxonomic units (OTU) were grouped at 97 % sequence similarity (approximately equivalent to genus level).

### Nitrous oxide emissions from in soil

N_2_O emission measurements were made from three replicate plots of each of the 9 treatments over the period October 2008 to August 2009. Emissions were measured using the static chamber technique (Mosier 1989), with three chambers (each covering 0.16 m^2^) randomly allocated per plot to account for spatial variability. Chambers were closed to allow headspace accumulation of N_2_O for 40 min before gas samples were taken from each chamber and stored in pre-evacuated vials (Chadwick et al. 2014). Initial chamber concentration was assumed to be the same as for ambient air, for which ten samples were taken on each sampling occasion at chamber height. Linearity of headspace accumulation of N_2_O was confirmed by taking additional samples from selected chambers at 20 and 60 min after closure. Emission measurements were made on a total of 32 occasions over the experimental period. Samples were always taken between 10 am and 12 pm. Gas samples were analysed as soon as possible after collection using gas chromatographs fitted with an electron-capture detector and an automated sample injection system. The N_2_O flux for each chamber at each sampling occasion was determined from the increase in headspace concentration. Cumulative emissions between two sampling occasions were calculated as the product of the mean plot flux for the two occasions and the time interval between. Data was expressed as g N_2_O-N g^−1^ dry soil d^−1^ (assuming that the upper 23 cm soil contains actively denitrifying bacteria) to allow direct comparison with microbial abundance and other properties expressed per g dry soil.

### Soil invertebrates

Mesofauna were extracted from three 8 cm dia., 10 cm deep soil cores taken from the grid areas assigned for soil sampling in each plot and identified as described previously (Hirsch et al. 2009).

### Soil pH, carbon and nitrogen

Soil pH was measured using a standard procedure where replicate 10 g aliquots of air-dried soil were suspended in 25 ml freshly-boiled deionised water. Soil organic carbon (SOC) and total N was determined by LECO analysis, subtracting carbonate released by incubation with hydrochloric acid from total C to estimate organic C. To extract soluble C from soil (Ghani et al. 2003), samples frozen immediately after collection and stored at −80 °C, were shaken in sterile distilled H_2_O (sd H_2_O - soil: water, 1: 10) at 300 rpm for 30 min, 20 °C. After centrifugation (swing-out rotor, 246×*g*, 20 min, 20 °C) the supernatant was decanted, soil resuspended and shaken as before for 16 h at 80 °C. The total non-purgeable organic C in the supernatant of this fraction was determined by wet oxidation using a Shimadzu TOC-V WS analyser. A simplified method was used to measure un-decomposed organic detritus (from plants and mesofauna): 5 g soil suspended in 10 ml sdH_2_O was shaken for 24 h at 300 rpm, 4 °C. The suspension was centrifuged (swing-out rotor, 50×*g*, 10 min, 20 °C) and the material remaining in suspension was weighed after collection on pre-weighed Whatman no 1 filters and overnight drying at 80 °C.

### Statistical methods

Statistical analysis was done using one-way ANOVA in GenStat 17th Edition (VSN International Ltd., Hemel Hempstead, UK). To check that each set of measured values met the assumptions of ANOVA and were normally distributed, residuals were plotted. If they did not show normal distribution, data was log-transformed and again checked for normal distribution of residuals. Where ANOVA results were significantly different (*p <* .05), means were further tested using Tukey’s post-hoc method in the GenStat multiple comparison menu with 95 % confidence; significantly different means are considered to have α = 0.05 and are referred to as “significant” throughout the text. Microbial diversity data, i.e. the relative abundance of OTU in each plot, was subjected to multivariate analysis non-metric multidimensional scaling (NM-MDS) based on a Bray-Curtis matrix, using the statistics analysis package PAST (Hammer et al. 2001). PAST was also used to estimate diversity indices, and to test the significance between communities in the different treatments using two-way ANOSIM and SIMPER.

## Results

### Soil properties

The soil pH did not show significant differences between plots and years, with a mean pH of 6.5. Wheat yields fluctuated each year but in the 4 years following conversion, compared to the permanent arable plots, grain and straw yields were consistently higher in conversions from grass and lower in conversions from bare fallow. The mean dry weights of grain in the grassland and bare fallow conversions, and permanent arable plots were respectively 5.0, 2.0 and 6.5 t ha^−1^, all significantly different (*F*_2,52_ = 33, *p* < .001). The corresponding values for straw were 2.7, 1.6 and 4.2 t ha^−1^, significantly higher in the grass to arable conversions (*F*_2,52_ = 14.76, *p* < .001).

**Fig. 1:**
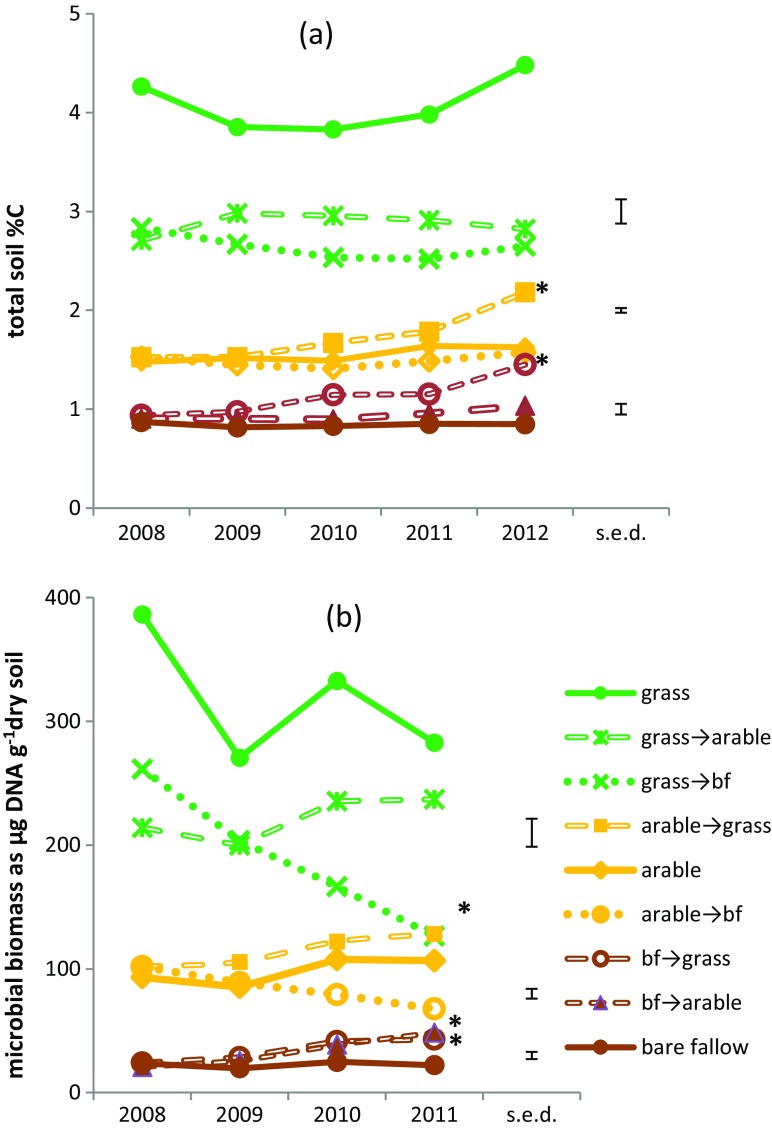
Soil organic C and microbial biomass in conversion experiment: (**a**) total % C for soil collected in October at the start and during first 4 years of conversion, measured by Leco combustion analysis - one-way ANOVA (*F*_8, 126_ = 354.54; *p* < .001) showed significant differences according to the original permanent treatments; *denotes significant increase in conversion compared to the corresponding permanent treatment in 2012; (**b**) total DNA yields from soils as a proxy for microbial biomass, collected in October at the start, and during first 3 years of conversion - one-way ANOVA (*F*_8, 81_ = 107.75; *p* < .001) showed significant differences according to the original permanent treatments; *denotes significant increase in conversion compared to the corresponding permanent treatment in 2011. Standard errors of differences of means (s.e.d.) calculated for each set of plots derived from the same original treatment are shown; *bf* bare fallow

Total SOC and N (%C and %N) were measured in October for the first 4 years of conversion. At the time of sampling, most fertilizer N added to the arable plots would have been assimilated by the crop and increases due to this source are neither predicted nor observed. Figure 1a shows the upward trend in SOC in the bare fallow plots converted to arable or grassland management and the arable plot converted to grassland and this was statistically significant for conversions to grassland by 2012. Soil N was approximately 10 % of soil C in all treatments and showed the same trends, indicating it was derived from the soil organic matter where C: N is typically 10: 1. In the first year after conversion, plots that were originally grassland showed a rapid drop of around 35 % in SOC and N when the soil was ploughed, remaining at this level for the subsequent 4 years; the regularly tilled arable and bare fallow plots did not show an initial drop. Compared to the initial grassland, SOC had fallen by 80 % 50 years after the original conversion to bare fallow and 65 % after conversion to arable. The yields of soil-extracted DNA, which correlate with soil microbial biomass (Hirsch et al. 2009) collected in 2008, 2009, 2010 and 2011 show a similar trend to total soil %C with a significant drop by 2011 where grassland or arable was converted to bare fallow, and significant increases for bare fallow conversions to arable and grassland (Fig. 1b). The permanent grassland soil gave inconsistent, highly variable DNA yields, albeit significantly more than in the conversion to the alternative treatments, possibly due to accumulated plant and mesofaunal material making it difficult to equate DNA yield with microbial biomass. Three years after conversion, in 2011, the previous management of each plot determined the size of the bacterial community as 16S rRNA gene copies g^−1^ dry soil, using real-time qPCR in the order bare fallow < arable < grassland (Table 2). There were significant increases in bacterial abundance where bare fallow was converted to arable or grassland, and a significant drop where grassland was converted to bare fallow, in agreement with the DNA yields.

**Table 2:** Abundance of 16S rRNA, denitrification and nitrification genes assessed by qPCR in each soil treatment in October 2010 (below each mean value is the standard error of the mean and the number of replicates)

	gene copies g^−1^ dry soil (x 10^−6^)					
	16S	*nirS*	*nirK*	*nosZ*	*amoA-*B	*amoA-*A
bare fallow	306.25 (18.24, n8)	0.16 (0.04, n7)	0.64 (0.03, n7)	0.69 (0.10, n7)	0.68 (0.32, n7)	0.43 (0.12, n7)
bf → arable	**616.16** (77.01, n7)	**0.52** (0.12, n7)	**1.80** (0.18, n7)	**2.12** (0.42, n7)	**2.44** (0.75, n7)	1.40 (0.40, n8)
bf → grass	**614.29** (71.47, n6)	**0.63** (0.63, n6)	**2.84** (0.46, n6)	**2.20** (0.38, n6)	0.20 (0.07, n6)	0.56 (0.15, n7)
arable	1380.78 (61.85, n7)	0.54 (0.06, n8)	8.64 (0.34, n7)	6.10 (0.28, n8)	5.01 (0.41, n8)	5.17 (0.47, n8)
arable→bf	991.73 (27.93, n7)	0.45 (0.08, n11)	7.94 (1.33, n12)	6.89 (1.13, n11)	1.97 (0.34, n8)	4.76 (0.72, n8)
arable→grass	1613.08 (83.01, n8)	0.92 (0.08, n8)	**11.55** (0.92, n8)	6.62 (0.40, n7)	2.77 (0.33, n8)	4.47 (0.82, n10)
grass	3373.52 (138.58, n6)	1.06 (0.14, n7)	36.81 (1.23, n7)	28.07 (0.77, n7)	0.12 (0.02, n7)	0.83 (0.18, n14)
grass→bf	**2134.71** (112.36, n9)	**0.44** (0.06, n6)	**12.88** (0.58, n6)	**11.07** (0.53, n6)	**3.08** (0.56, n8)	**2.47** (0.29, n15
grass→arable	2972.48 (127.95, n7)	0.81 (0.11, n7)	**23.38** (0.69, n7)	21.35 (2.64, n8)	**4.01** (0.55, n7)	**3.64** (0.54, n7)
ANOVA	*F*_8,56_ = 111.97 *P* < .001	*F*_8,58_ = 11.96 *P* < .001	*F*_8,58_ = 137.53 *P* < .001	*F*_8,58_ = 65.81 *P* < .001	*F*_8,57_ = 28.83 *P* < .001	*F*_8,75_ = 12.29 *P* < .001

Gene copy numbers were log-transformed to meet assumptions for ANOVA (untransformed values shown). Post-hoc comparisons of means significantly different from the corresponding permanent treatment are indicated in bold

*bf* bare fallow

Labile organic C extracted using hot water was least in the bare fallow and most plentiful in the grassland soils with significant increases in the bare fallow plots converted to arable and grassland and the arable plots converted to grassland by 2012 (Table 3). A similar trend was apparent for labile soil C in 2010 but only the increase in bare fallow plots converted to grass was significant. In 2012, significantly more light detritus (mostly of plant origin on examination using a light microscope) was extracted only from the permanent grassland compared to the other plots (*F*_8,18_ = 10.57, *p* = <.001).

**Table 3:** Labile carbon extracted with hot water in October 2012

Mean μg C g^−1^ dry soil	
bare fallow	32.3
bf→arable	**44.7**
bf→grass	**77.0**
arable	69.5
arable→bf	65.9
arable→grass	**116.1**
grass	183.4
grass→bf	156.0
grass→arable	197.5

Values were log-transformed to meet assumptions for ANOVA (labile C, *F*_8,17_ = 22.11, *p* = <.001; values in bold are significantly different from the corresponding permanent treatments

*bf* bare fallow

### N_2_O emissions

The N_2_O emissions were highly variable throughout the year although the fortnightly data indicated higher emissions in the month following conversion which may be due to warm, wet conditions in October, more conducive to denitrification. The mean daily N_2_O emissions were significantly increased during the year after conversion in the arable to bare fallow and grassland to arable or bare fallow conversions (Supplementary Fig. 3).

### Nitrogen cycling genes

Two years after conversion, in October 2010, the abundance of genes involved in denitrification and nitrification was estimated using qPCR (Table 2). The estimated number g^−1^ dry soil of *nirS, nirK* and *nosZ* varied significantly in all three permanent treatments increasing in the order bare fallow < arable < grass, a similar trend to the total prokaryote community assessed from the 16S rRNA gene copy number. All showed increases where bare fallow was converted to arable or grass and where arable was converted to grass, and decreases where grassland was converted to either bare fallow or arable, the same trends as were seen for the bacterial 16S rRNA gene. Abundance of the nitrous oxide reductase gene *nosZ* was similar to that of *nirK,* with the alternative nitrite reductase *nirS* at least 10 times less common, similar to the relative abundance observed previously in other arable soils at Rothamsted (Clark et al. 2012).

Abundance of bacterial and archaeal genes for ammonia monooxygenase (*amoA–*B; *amoA-*A copies g^−1^ dry soil) was similar in comparable plots, with both groups significantly more numerous in the permanent arable plots that received both fertilizer N and tillage than in the permanent grassland or bare fallow (Table 2). There was a significant increase in *amoA*-B compared to the corresponding permanent plots where bare fallow was converted to arable and for both groups where grassland was converted to arable or bare fallow.

### Mesofauna

**Fig. 2:**
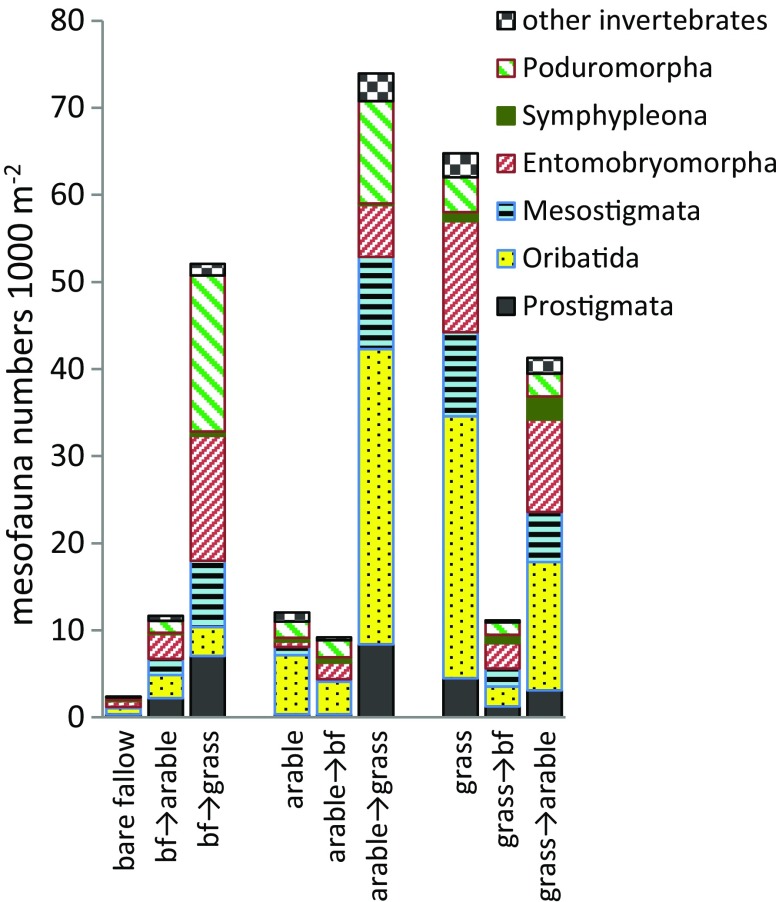
Invertebrate counts in soil sampled in October 2010. *bf* bare fallow. The Prostigmata, Oribatida and Mesostigmata are mites; Entomobryomorpha, Symphyphleona and Poduromorpha are collembola. Total numbers of mites, collembola and all mesofauna were log-transformed to meet assumptions for ANOVA; total mesofauna (*F*_8, 71_ = 18.11, *p* < .001) and the number of mites (F_8, 71_ = 16.18, *p* < .001) were significantly different in all permanent plots, the number of collembola (F_8, 68_ = 10.56, *p* < .001) was significantly higher in permanent grass than in bare fallow, increasing significantly when bare fallow and arable were converted to grass

Two years after conversion, permanent grass hosted 5-fold more mesofauna than the arable soil which in turn held 5-fold more than the bare fallow (Fig. 2). This difference in the permanent treatments had been reported previously (Hirsch et al. 2009). Total numbers of all mesofauna including mites and collembola had increased significantly in plots where bare fallow or arable was converted to grass and decreased significantly where grass was converted to bare fallow. Although there were fewer mesofauna where plant inputs were reduced in conversions to bare fallow or arable, 2 years later they had not fallen to the very low numbers seen in permanent bare fallow soil.

### Bacterial, archaeal and fungal diversity

Changes in archaeal and bacterial community structure were investigated 3 years post conversion, in 2011, using methods based on variation in the diversity and relative abundance of 16S rRNA gene amplicons. The mean number of OTUs, and diversity indices based on OTU “richness” (Chao1, Fisher’s alpha), were not significantly different between plots. However, Shannon’s *H’* which reflects how OTU are distributed between samples, indicated significantly less “evenness” in permanent grassland and grassland-derived plots compared to the other treatments (*F*_8,18_ = 31.14, *p* < .001). The Simpson indices for evenness and dominance supported this, showing significant differences in plots derived from all three permanent treatments in order of decreasing evenness bare fallow > arable > grassland (*F*_8,18_ = 46.93, *p* < .001).

**Fig. 3:**
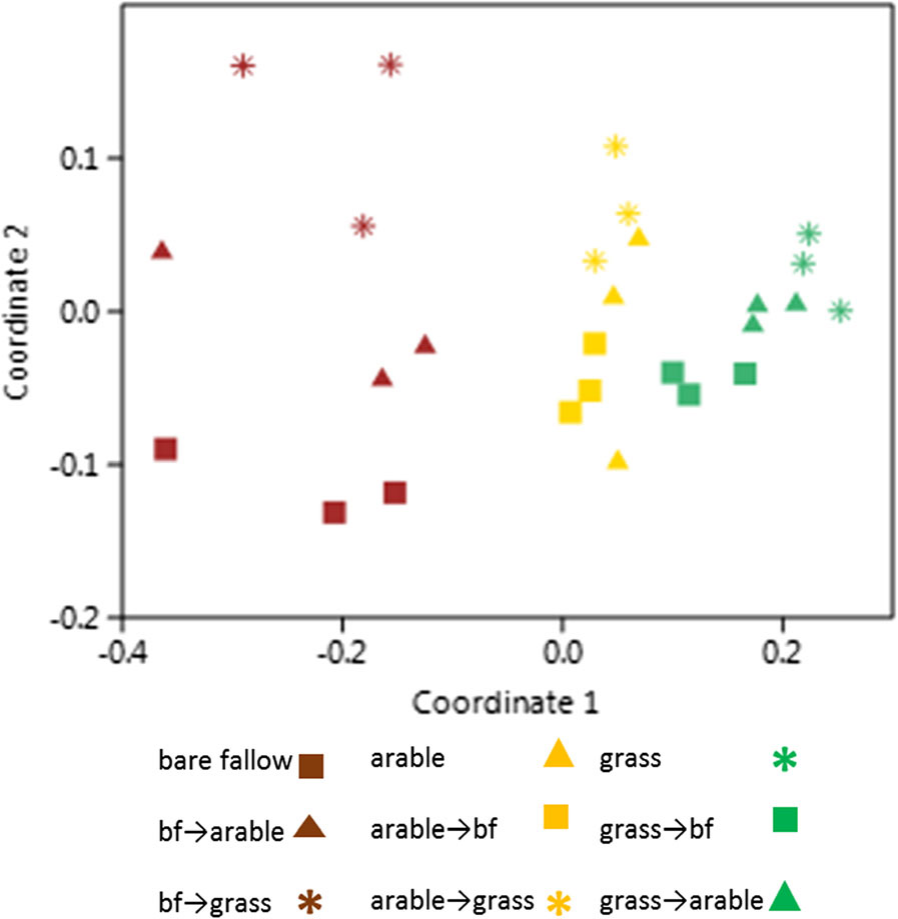
Non-metric MDS (stress = 0.08) comparing the presence and relative abundance of 16S rRNA amplicons identified to OTU level, soil collected in 2011, *bf* bare fallow

Non-metric MDS at OTU level shows separation of communities according to the original plot treatment (Fig. 3). A two-way ANOSIM test of the data showed that the grouping according to the original treatment (*R* = 0.85, *p* = .0001) is more significant that the current treatment (*R* = 0.61, *p* = .0001). When OTUs for samples taken in 2008 prior to conversion were compared to those from 2011 (permanent treatments only), there was separation according to both treatment and year (MDS not shown; ANOSIM for treatment *R* = 0.83, *p* = .0001; for year *R* = 0.83, *p* = .0008).

SIMPER analysis of the 17 prokaryotic Phyla (subphyla for the Proteobacteria) contributing > 1 % to the differences between treatments, cumulatively 99.3 %, is shown in Table 4. The Verrucomicrobia contributed most: they were the most abundant in permanent arable and grassland plots and differed significantly in order of relative abundance grassland > arable > bare fallow; there was also a significant reduction where grassland was converted to bare fallow. The Gemmatimonadetes (ranked 3rd) showed a converse distribution, most abundant in the bare fallow and least in the grassland, with significant reductions where bare fallow was converted to arable or grassland. The β-Proteobacteria (ranked 2nd) and Armatimonadetes (17th) were also most abundant in bare fallow; Planctomyces (12th) were most abundant in grassland. There were significantly fewer α-Proteobacteria (5th), Choloroflexi (8th), Cyanobacteria (14th) and Chrenarchaeota (16th) in grassland soil compared to the other permanent treatments. The Crenarchaeota (in our soils, represented by the class Thaumarchaeota, order Nitrososphaerales, ammonia-oxidizing archaea) were most abundant in the arable plots and those derived from them, in agreement with the qPCR results for archaeal *amoA*. The nitrifiying phylum Nitrospirae was also significantly more abundant in the arable soil.

**Table 4:** SIMPER analysis of October 2011 16S amplicon data at Phylum level (sub-phylum for the Proteobacteria) ranking the groups contributing more than 1 % of the differences observed, showing the relative abundance (mean % amplicons in each treatment) for each group

Phylum or Subphylum	SIMPER % contributed/ culmulative		Bare fallow (bf) converted to			Arable (ar) converted to			Grass (gr) converted to		
			bf	ar	gr	ar	bf	gr	gr	bf	ar
Verrucomicrobia	31.8	31.8	**11.30**	10.70	10.90	**20.10**	20.00	19.00	**31.10**	25.10	28.10
β-Proteobacteria	9.8	41.6	**10.60**	8.71	7.33	4.79	5.00	4.42	3.33	4.44	3.81
Gemmatimonadetes	7.6	49.1	**7.50**	4.87	4.34	**3.34**	3.81	2.28	**1.41**	2.88	1.74
Actinobacteria	7.3	56.5	9.90	10.50	13.30	9.75	11.80	13.70	9.96	11.60	10.20
α-Proteobacteria	6.4	62.8	12.20	14.00	14.60	13.40	12.70	13.20	**10.20**	12.20	11.00
Acidobacteria	6.2	69.0	16.30	16.40	16.80	17.20	14.40	15.20	15.00	13.70	15.30
Firmicutes	4.9	73.8	2.97	3.94	3.38	1.64	3.39	2.11	0.91	2.35	1.60
Chloroflexi	3.9	77.7	3.92	4.12	4.93	3.82	2.93	3.95	**2.26**	2.65	2.63
δ-Proteobacteria	3.5	81.2	5.45	5.33	4.96	5.77	5.83	5.68	6.00	5.70	7.23
Bacteroidetes	3.3	84.5	5.47	5.82	6.12	4.34	4.75	5.98	5.51	4.96	4.18
γ-Proteobacteria	3.1	87.6	3.43	4.83	3.62	4.35	4.06	3.98	3.27	4.64	3.93
Planctomycetes	3.0	90.6	4.71	4.32	5.37	5.28	5.22	5.46	**7.00**	5.03	5.63
Nitrospirae	2.3	92.9	1.27	1.45	0.44	**2.15**	1.52	1.75	1.21	1.47	1.79
Cyanobacteria	2.0	94.9	1.33	1.71	0.64	0.70	0.84	0.36	**0.23**	0.56	0.34
WS3 Latescibacteria	1.8	96.7	0.79	0.54	0.45	1.59	1.28	0.91	0.66	0.83	1.03
Crenarchaeota	1.4	98.0	0.51	0.69	0.23	0.77	1.02	0.60	**0.06**	0.44	0.27
Armatimonadetes	1.3	99.3	**0.97**	0.73	0.71	0.48	0.62	0.36	0.09	0.24	0.17

Post-hoc comparisons of means significantly different in the permanent treatment are indicated in bold; significantly different means within groups compared to the corresponding permanent treatment are underlined

A more detailed SIMPER analysis (Supplementary Table 2) showed that 16 OTU contributed 1 % or more to the total difference between treatments, cumulatively 39.5 %. Verrucomicrobia related to genus DA101 were most influential, the distribution similar to that of the Phylum (Table 1); an OTU assigned to the δ-Proteobacteria family Syntrophobactereaceae was likewise significantly more abundant in grassland. Two OTU assigned to β-Proteobacteria orders were significantly more abundant in plots originating from bare fallow as were OTU belonging to Gemmatimonadetes. The difference between looking at phyla and OTU is exemplified by the α-Proteobacteria: this sub-phylum was least abundant in grassland, most abundant in bare fallow soil as was an OTU assigned to the genus *Kaistobacter.* However, a second α-Proteobacteria OTU assigned to *Rhodoplanes* was significantly less abundant in bare fallow soil.

SIMPER analysis of OTU from 2008 were compared with those from 2011 in the permanent plots (Supplementary Table 3). As in the other analyses, the most influential OTU were assigned to Verrucomicrobia DA101, significantly more abundant in grassland soil in 2011 compared with 2008, less abundant in bare fallow soil in 2008. Similar, apparently arbitrary changes between the years are seen for other OTU, a warning against over-interpretation of SIMPER results.

**Fig. 4:**
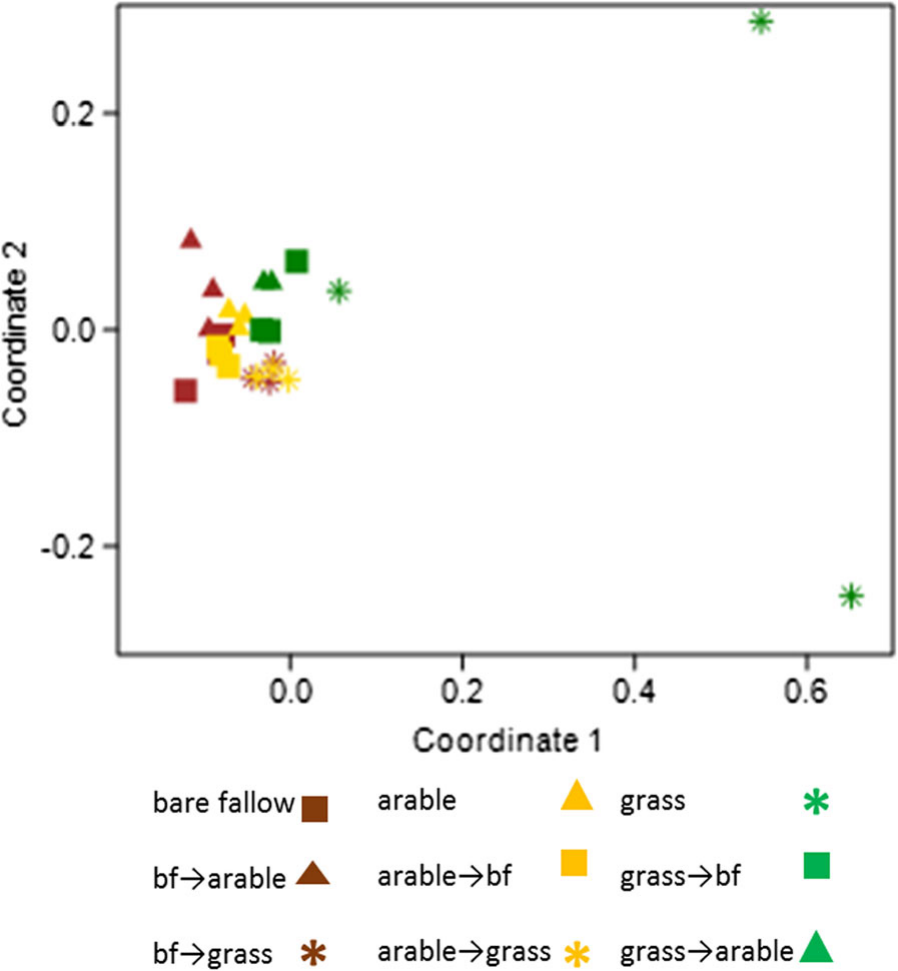
Non-metric MDS (stress = 0.24) of fungal community ITS profiles at OTU level, soil collected in 2011, *bf* bare fallow

Fungal NM-MDS based on ITS (Fig. 4) indicates the greatest difference between the communities in permanent grassland, the other treatments appearing to cluster. Two-way AONSIM indicates that differences according to the current treatments are more significant (*R* = 0.68, *p* = .0001) than the plot origin (*R* = 0.47, *p* = .0001). The 24 fungal groups that contribute ≥ 1 % of the differences between treatments, cumulatively 49.8 %, are shown in Supplementary Table 4. The majority of groups were Ascomycota (15 OTU) followed by Basidiomycota (6 OTU) and Zygomycota (3 OTU) with one group identified only to the Fungi. Only 7 of these OTU could be identified to genus level. An Ascomycete assigned to *Gibberella* (anamorph of the cereal pathogen *Fusarium*) was most abundant in arable soil, several Basidiomycete OTU of the class Agaricomycetes (wood-decaying fungi) were significantly more abundant in grassland soil and an unidentified Ascomycota OTU was most abundant in bare fallow soil. Saprophytic Ascomycota assigned to *Cladosporium* and Basidiomycota assigned to the class Tremellomycetes were significantly less abundant in the bare fallow soil.

## Discussion

The plots with contrasting treatments now considered “permanent” had developed distinctly different properties after 50–60 years, notably the loss of soil aggregate structure and SOC in bare fallow soil compared to the original grassland (Hirsch et al. 2009; Watts et al. 2001). Increased crop yields in soils converted from long-term grassland to arable use has been observed before and is often attributed to the absence of deleterious soil organisms including the take-all fungus *Gaeumannomyces graminis,* together with increased N arising from decomposition of soil organic matter. The previously bare-fallow plots yielded significantly less than the permanent arable so presumably other factors including the poor soil structure related to low SOC affected yields.

Despite the limited number of samples that it was practical to analyze, changes in soil C, N and biotic factors were statistically significant following the conversions. The rapid drop of around 35 % in SOC and N when grassland soil was ploughed was probably an artefact due to burial of SOC-rich topsoil at 23 cm, below our 10 cm sampling depth. Subsequent ploughing will partially reverse this, especially in the multiply-tilled bare fallow plots although it may also accelerate SOC degradation. There is no record of SOC immediately after the original conversion from permanent grassland to bare fallow but a reduction of around 21 % was reported after 4 years (Coleman et al. 1997). Mesofauna and DNA yields which approximate to the microbial biomass, were both on a significant downwards trajectory in the grassland to bare fallow conversions. There were significant increases in the abundance of mesofauna and soil bacteria g^−1^ dry soil (according to both 16S rRNA gene copy number and DNA yield) where bare fallow plots were planted with either wheat or grass. The relationship between plant cover and microbial biomass is well known but mostly compares chronosequences (Jangid et al. 2011; de Vries et al. 2013) rather than samples taken during the early stages of conversion.

In the regularly tilled arable and bare fallow plots, SOC (and soil-extracted DNA) remained constant. We assume that in the bare fallow plots, there was some C input from sporadic weeds prior to their removal, and by phototrophic microorganisms. Where bare fallow or arable plots were converted to grass, SOC had increased significantly after 4 years, by 71 and 35 %, respectively. Further investigation indicated this was not only due to plant and other organic detritus which increased significantly in these plots, but also to increases in labile C. This labile C is assumed to include microbial and plant-derived material; the light detritus also increased significantly where bare fallow plots were converted to arable confirming the trend seen in total SOC and soil biota in these plots. We measured 70 μg labile C g^−1^ dry soil in the arable plots, of similar magnitude to the “extractable organic C” in the upper 10 cm of wheat soil reported by Kramer et al. (2013). Different C fractions have been described for plant inputs to soil (Coleman et al. 1997); for Rothamsted soils, measured and modelled total inputs are estimated to be 4–5 T C ha^−1^ y^−1^ from grass and 1.4 T C ha^−1^ y^−1^ from arable cropping into the top 23 cm soil (Johnston et al. 2009). We measured a similar input: the total increase where bare fallow plots were converted to grassland was 5 mg SOC g^−1^ dry soil after 4 years, equivalent to 4 T C ha^−1^ y^−1^. The relatively low numbers of soil mesofauna and microorganisms present at the onset of the conversion may allow more SOC to accumulate in the bare fallow conversions during the early stages and it will be interesting to observe if the rate of accumulation declines in subsequent years.

A consequence of increased availability of plant material in conversions to grassland was a rapid increase in mesofauna numbers, with detritivorous collembola (Poduromorpha, Entomobryomorpha) and mites increasing significantly after 2 years. The mites included predators (Mesostigmata), plant feeders (Prostigmata) and detritovores (Oribatida), indicating that a plant-based food web was established. Conversion to bare fallow resulted in a reduction in all groups, including the detritivores. Complex interactions influence population dynamics (Van der Putten et al. 2001), but a similarly rapid increase in soil mesofauna has been reported in mine restoration projects (Andres and Mateos 2006).

As with the mesofauna, the relative abundance of genes involved in cycling nitrogen, and by association the bacteria that carry them, were shown to have changed after 2 years of conversion. The dominant nitrite reductase gene (*nirK*) and nitrous oxide reductase (*nosZ*) were most abundant in the permanent grassland and least abundant in the permanent bare fallow plots and the influence of the original treatment was apparent in the conversions although the positive influence of grassland on bacteria carrying *nirK* is indicated by the significant increase where bare fallow was converted to grassland and decrease where grassland was converted to bare fallow or arable. The highest mean daily N_2_O emissions measured during the preceding year were in the grass to bare fallow or arable conversions, and the arable to bare fallow. In the former, the upper layer of grassland soil with an abundant nitrifier and denitrifier population and C- and N-rich organic matter was disrupted and buried; the latter received wheat stubble and roots. Thus, the addition of N fertilizer to permanent arable plots did not appear to disproportionally favour bacteria carrying genes for denitrification or to result in increased N_2_O emissions. Rather, the increased N_2_O emissions observed during the first year in the grassland conversions to bare fallow and arable, and arable conversion to bare fallow, indicate that the consequences of buried SOC and the associated organic N are initially more important than applied N fertilizer for increasing denitrification activity. This reinforces concerns that conversion from grassland to arable cropping increases greenhouse gas emissions (Six 2013). Fungal denitrification is reported to contribute to N_2_O emissions in arable soil (Herold et al. 2012) but our results showing increases following physical perturbation indicate that fungal activity is unlikely to have played a major role.

Similarly, although most ammonia oxidizing bacteria and archaea are capable of denitrification, they are considered unlikely to contribute significantly in agricultural soils dominated by heterotrophic denitrifying bacteria. Their key role in the N cycle is producing nitrite for the second step in nitrification: the abundance estimated from their specific *amoA* genes g^−1^ dry soil in permanent arable plots was significantly greater than in either bare fallow or grassland. This is presumably due to a lack of substrate (no fertilizer ammonia added, in contrast to the arable treatments) with an additional effect due to the selective disadvantage of the archaea in the undisturbed grassland soil (Zhalnina et al. 2013). Conversion of arable plots to bare fallow or grassland, where no fertilizer was applied, resulted in a decrease in ammonia-oxidizing bacteria; they increased significantly when bare fallow or grassland plots were converted to arable. These changes were apparent in 2010, 2 years after conversion, despite reports that both AOA and AOB are relatively slow growing (Prosser and Nicol 2012). Results iindicate that where ammonia is added as a fertilizer, the bacteria and archaea that use it as an energy source are more abundant than in unfertilized plots. The archaeal ammonia oxidizers were identified by SIMPER as one of the drivers of the differences in multivariate analysis, as were the Nitrospirae, a phylum involved in nitrite oxidation. Despite some divergence in the multivariate analyses, overall, the relative abundance of different species of bacteria and archaea (assessed by 16S rRNA gene amplicon sequencing) had not undergone dramatic changes 3 years after conversion, with the prokaryotic community structure most closely resembling the original treatments in 2011. A gross temporal shift was also evident over this period for the permanent treatments. In a different study, we observed temporal differences in metagenomic samples taken in different months and years (Delmont et al. 2012). The Highfield conversion soil was sampled in both 2008 and 2011 by the same team during the first 2 weeks of October and there were no significant differences in weather patterns prior to sampling. We assume that this shift is due either to subtle differences in environment, or it represents stochastic changes in the relative abundance of members of the microbial communities over time, as reported by Lauber et al. (2013).

The diversity indices imply that certain prokaryotic OTU dominate the grassland plots whereas they are more evenly distributed in disturbed bare fallow plots (with arable plots intermediate). This may reflect the lack of plants that provide a selective advantage to certain groups in bare fallow soil and the ubiquity of roots in the grassland soil. Many of the same groups (generally, the most abundant) were implicated by SIMPER analysis to contribute to the differences between both treatments and years. The most influential bacterial group according to SIMPER analysis, most abundant in grassland and least in bare fallow soil, was assigned to the Verrucomicrobia group DA101, belonging to the order Chthoniobacterales which is represented by a cultured isolate, *Chthoniobacter flavus*. The Chthoniobacterales are very common in soil (Sangwan et al. 2004) but little is known of their biology. *C. flavus* is reported to grow on sugars, sugar polymers and pyruvate and to be unable to grow anaerobically by denitrification. This would be consistent with rapid responses to changes in the availability of substrates and oxygen in soil and a smaller population where less substrate is available. The Gemmatimonadetes, a prevalent group most abundant in bare fallow soil, are reported to have an advantage in arid conditions (DeBruyn et al. 2011). However, phyla and sub-phyla contain many different groups which may have contrasting requirements, exemplified by the α-Proteobacteria *Kaistobacter* and *Rhodoplanes:* the former appears to favour plant-free bare fallow soil, the latter is more abundant in grassland. The higher relative abundance of the nitrite-oxidizing phylum Nitrospirae and the ammonia-oxidizing Chrenarchaeota in arable soil indicates that these groups benefit from the annual addition of N fertilizer to the arable plots.

Compared to the prokaryotes, it was more difficult to distinguish difference in fungal communities in the treatments with the exception of two permanent grassland plots. These did not cluster in multivariate analyses although conversions from grassland clustered tightly with the other treatments. SIMPER analysis did reveal some OTU associated with each of the three permanent treatments, possibly explained by differing lifestyles, as cereal pathogens or saprophytes. Differences in the drivers of fungal and bacterial diversity have been reported recently (Hogberg et al. 2014). The fungal OTU richness was similar in all plots and this is consistent with reports that plant diversity does not lead to fungal species diversity in soil (Johnson et al. 2010). The variation between replicate plots in the permanent grassland soils may be a consequence of the stochastic development of fungal communities in the untilled plots in association with roots over time, as observed in a recent analysis of fungal communities across different sites and spatial scales in Australia (Beck et al. 2015). The authors concluded that in contrast to this neutral (stochastic) assembly of rhizosphere fungi, communities associated with bulk soil, equivalent to our arable and bare fallow plots, were selected by environmental factors, defined as niche assembly. Fungal communities in conversions to grassland grouped closely indicating that the development of divergent assemblages is not a rapid process.

The absence of physical perturbation in the grassland plots is likely to contribute to the stochastic development of fungal communities and to the less even distribution of prokaryotic OTU, compared to the arable plots. Similarly, the repeated tilling of the bare fallow plots may explain their more even OTU distribution compared to the arable plots, as well as accelerating SOC degradation. Thus, the differences in tillage intensity in the Highfield experiment are an integral part of the three treatments alongside the presence or absence of plants.

## Conclusions

This experiment provided a unique opportunity to measure changes in soil biology and chemistry over a 4-year time period, long in terms of most studies on soil microbial ecology but very short compared to many studies on soil properties such as SOC. The study benefitted from the availability of soils with the same ancestry (meadow prior to 1948), similar pH (a major factor known to influence microbial communities) and climatic conditions but with contrasting long-term treatments. There has been little previous work on the effects of planting arable or perennial crops in previously unplanted and degraded soil. This experiment showed the rapid increase in SOC arising from plants, and the associated return of mesofauna which are an essential part of nutrient cycling in soil, in the grassland conversions. It also demonstrated that soil microbial numbers had started to increase after 2 years and key groups such as nitrifiers and denitrifiers were responding to changes in management and the addition of N fertilizer. Prokaryote biodiversity overall changed more slowly than the specific subgroups responding to N additions or the mobile mesofauna. In contrast, fungal communities, distinctive in permanent grassland, became more similar to the other treatments after conversion. Importantly, after 4 years, soil C concentrations had risen significantly after planting grass or wheat in the bare fallow soils and it will be interesting to see how rapidly these return to the C levels present in the corresponding permanent plots. Finally, results confirm that soil biota is resilient and that plants can provide degraded soils with the potential for rapid recovery.

## Electronic supplementary material

Below is the link to the electronic supplementary material.

ESM 1 (DOCX 864 kb)
