# Supplementary material for: Soil resilience and recovery: rapid community responses to management changes
Source: Plant Soil. 2016 Sep 30;412(1):283–97. doi: 10.1007/s11104-016-3068-x (PMC7045894; doi:10.1007/s11104-016-3068-x)
Supplement: Supplementary file 1 — (DOCX 864 kb) [file 11104_2016_3068_MOESM1_ESM.docx]

**Supplementary information**

**PCR methods**

The abundances of bacterial 16S rRNA*,* bacterial *amoA*, archaeal *amoA*, *nirK*, *nirS* and *nosZ* genes in all the soil samples were quantified using real-time PCR as described in Clark et al. 2012 (Phil Trans. Roy. Soc. 367: 1235–1244). Optimized quantitative real-time PCR was performed using primers described in Supplementary Table 1 with an Applied Biosystems 7900HT Fast Real-Time PCR System in a 384 well format. PCR conditions in a 10 μL reaction mixture consisting of 10 ng DNA template, 5.0 μL QuantiTect SYBR green master mix (Qiagen), contained optimised primer concentrations (Supplementary Figure 1): 1μM for bacterial 16S rRNA and archaeal *amoA*, 1.5 μM for bacterial *amoA*, 5 μM for *nirK* and *nosZ*, 7.5 μM for *nirS*. Real-time amplification was performed using the following programs. For bacterial 16S rRNA gene: initial 15 min at 95°C; 30 cycles of 15 s at 95°C for denaturing, 30 s at 58°C for annealing, 30 s at 78°C and 15 s at 84°C (both readout) for extension; for bacterial *amoA* gene: initial 15 min at 95°C; 45 cycles of 15 s at 95°C for denaturing, 30 s at 58°C for annealing, 45 s at 72°C and 15 s at 84°C (both readout) for extension; for archaeal *amoA* gene: initial 15 min at 95°C; 40 cycles of 15 s at 95°C for denaturing, 30 s at 58°C for annealing, 45 s at 72°C and 15 s at 78°C (both readout) for extension; for *nirK*, *nirS* and *nosZ* genes: initial 15 min at 95°C; 40 cycles of 15 s at 95°C for denaturing, 30 s at 58°C for annealing, 45 s at 72°C and 15 s at 84°C (both readout) for extension. Comparison of the two readout temperatures allowed monitoring for the production of primer dimers to ensure that they were kept at a minimum and did not affect either the efficiency of the reaction or the final quantification of the product.

In addition to ensure there were no detrimental effects of PCR inhibitors various DNA dilutions were tested. A melting curve analysis was performed to confirm PCR product specificity after amplification by measuring fluorescence continuously as the temperature increased from 50 to 95 °C. The specificity of the PCR was further evaluated by running randomly selected samples on a 1.5% (w/v) agarose gel.

Prior to qPCR, environment-specific standards for each primer set were generated. Serial dilutions of an aliquot of amplified product (generated from DNA extracted from pooled soil samples) were used with the relevant primer set to generate the standard curves for estimating gene copy number; the qPCR product was purified and quantified using QIAquick PCR Purification Kit (QIAGEN, Germany), Qubit^®^ dsDNA BR Assay kit (ThermoFisher Scientific) and a Qubit™ fluorometer (Invitrogen, NZ). This was to minimise the bias inherent in the usual approach, where one or only a few genes are used to standardize complex communities (Töwe *et al*., 2010 J. Microbiol. Meth. 82:338-341). Standards were diluted accordingly to give a concentration range from 0 to 10^7^ (10^9^ for bacterial 16S rRNA) gene copies μl^-1^. All DNA preparations were checked for the absence of inhibitors prior to PCR and all results were analysed using LinRegPCR program version 11.1 (Ramakers et al. 2003 Neurosci. Lett. 339:62-66.; Ruijter et al. 2009 Nucleic Acids Res 37: 1–12) to confirm the efficiency of amplification and the absence of inhibition. PCR standards are illustrated in Supplementary Figure 2.

The number of copies of each gene was calculated using the following equation:

gene copy number = (ng*number/mol)/(base pairs*ng/g*g mol base pairs) (http://www.uri.edu/research/gsc/resources/cndna.html).

**Supplementary Table 1** Primers used for quantitative PCR

| gene | primer | sequence | product (bp) | reference |
| --- | --- | --- | --- | --- |
| Bacterial16S | 331F | tcc tac ggg agg cag cag t | 194 | Nadkarni et al. (2002)  *Microbiology* 148: 257–266 |
|  | 534r | att acc gcg gct gct gg |  | Muyzer et al. (1993)  *Appl Environ Microbiol* 59**:** 695-700. |
| *nirK* | nirK876 | aty ggc ggv cay ggc ga | 165 | Henry et al. (2004)  *J. Microbiol Methods* 59: 327-335. (Erratum, 61: 289-290.) |
|  | nirK1040 | gcc tcg atc agr ttr tgg tt |  |  |
| *nirS* | nirS_cd3a-F | gts aac gts aag gar acs gg | 410 | Michotey et al. 2000  *Appl Environ Microbiol* 66: 1564-1571 |
|  | nirS_R3cd-R | gas ttc ggr tgs gtc ttg a |  | Throbäck et al. (2004)  *FEMS Microbiol Ecol* 49**:** 401-417 |
| *nosZ* | nosZ2R | cak rtg cak sgc rtg gca gaa | 268 | Henry et al. (2006)  *Appl Environ Microbiol* 72: 5181-51896 |
|  | nosZ2F | cgc rac ggc aas aag gts mss gt |  |  |
| Bacterial *amoA* | amoA-1F | ggg gtt tct act ggt ggt | 491 | Rotthauwe et al. (1997)  *Appl Environ Microbiol* 63: 4704–4712 |
|  | amoA-2R | ccc ctc kgs aaa gcc ttc ttc |  |  |
| Archaeal *amoA* | arch-amoAF | sta atg gtc tgg ctt aga cg | 635 | Francis et al. (2005)  *Proc Natl Acad Sci USA* 102: 14683–14688. |
|  | arch-amoAR | gcg gcc atc cat ctg tat gt |  |  |

**Supplementary Figure 1**

Example of PCR primer optimisation reactions - typical gel illustrating *nirK* PCR optimisation. Left-hand lane: 100 bp size marker; NTC - no template control; temperature gradient at 6 different temperatures , first set at primer concentrations 5000/5000 nmoles primer per reaction, followed by 7 different concentrations.


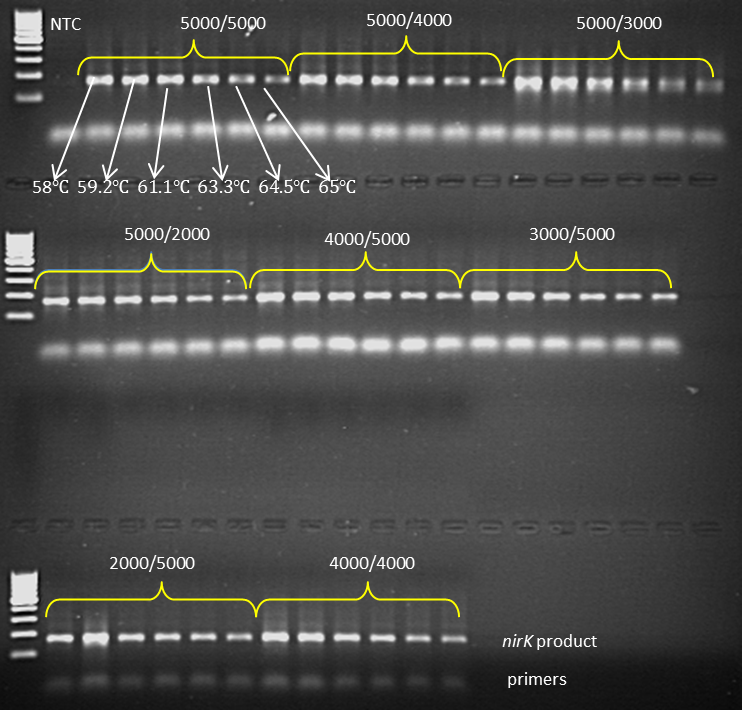


**Supplementary Figure 2**: Example of PCR standards. Control *nirK* PCR reactions: amplification plot on right with different known concentrations of target template; melt curve on left; standard curve generated from plot below


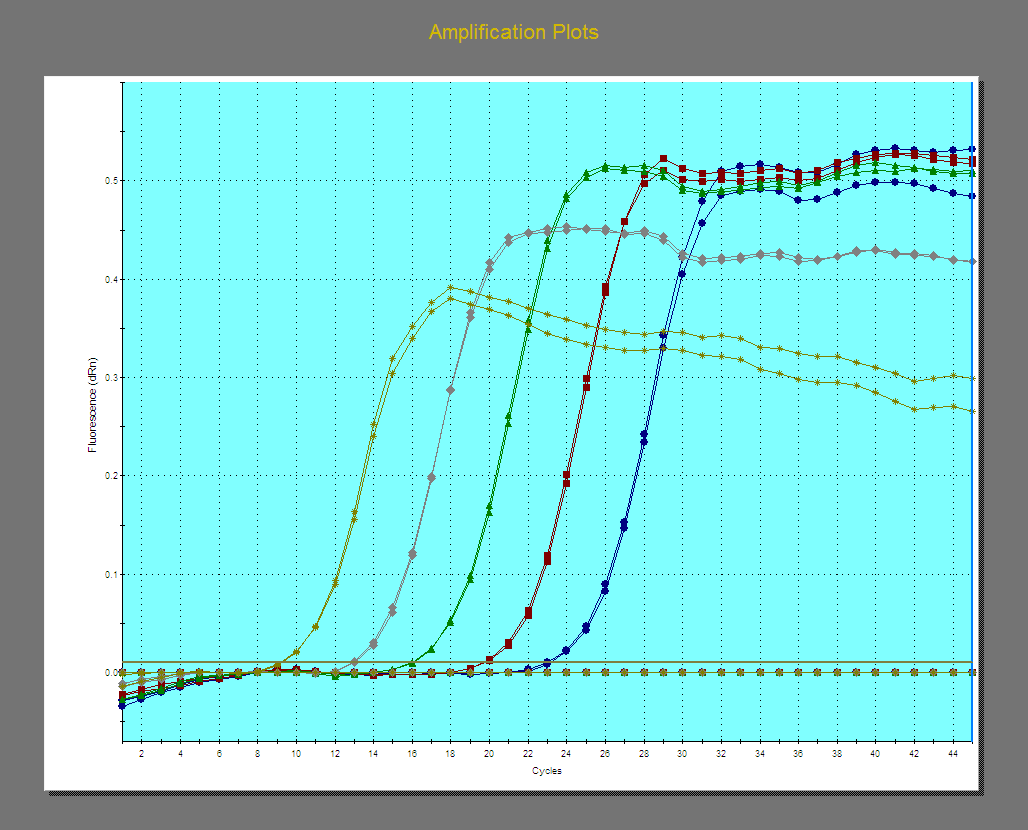

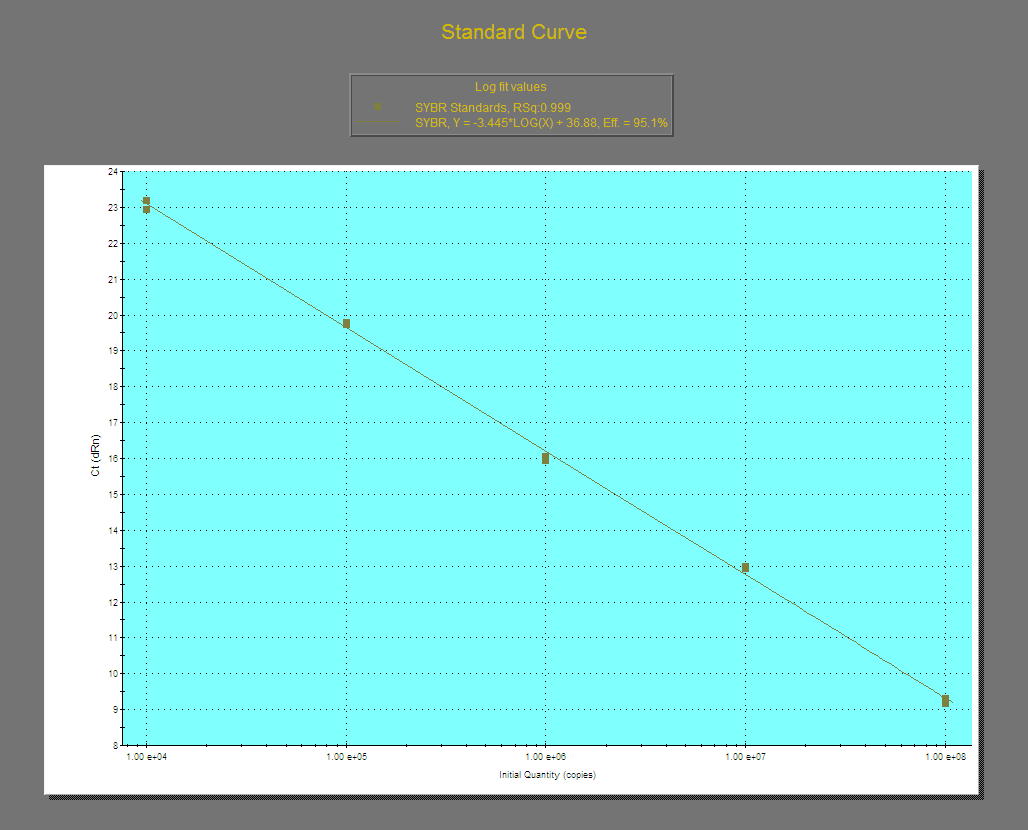

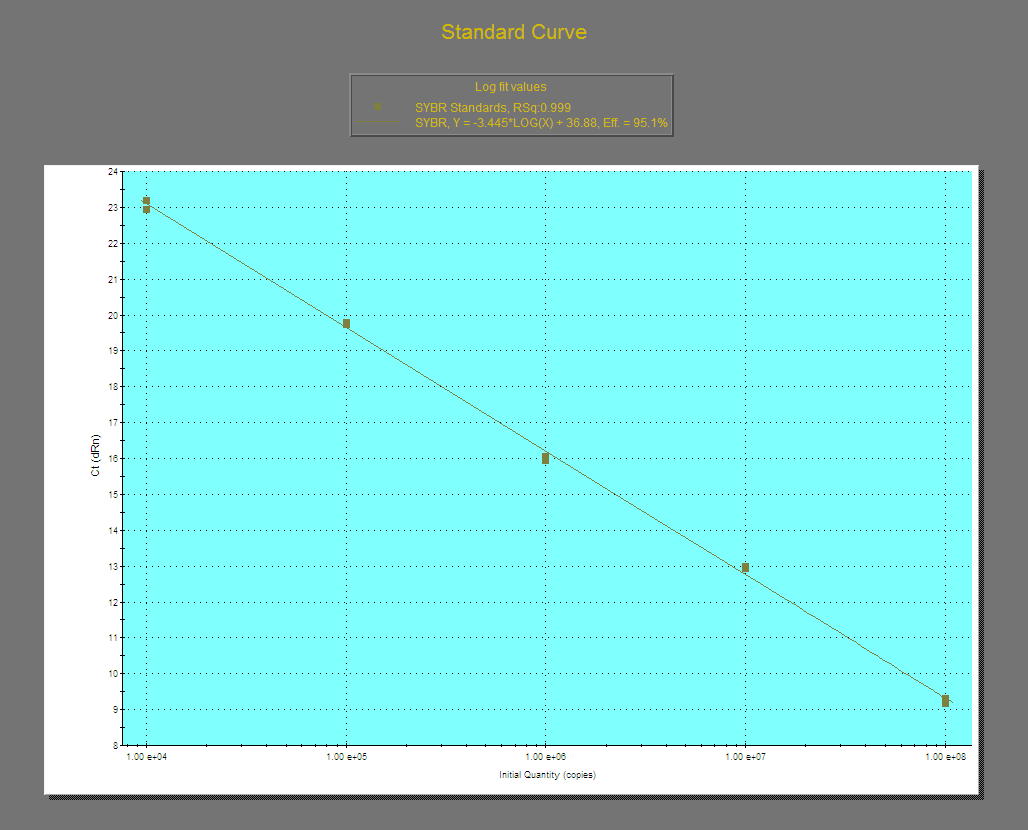

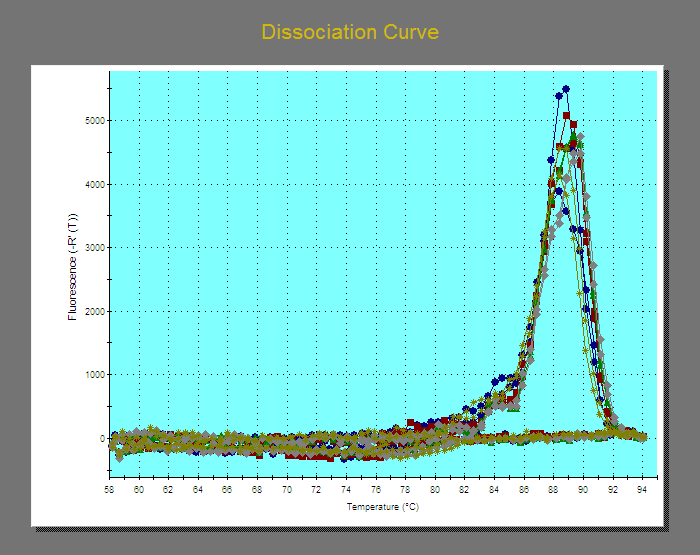


10^8^ copies

10^7^ copies

10^5^ copies

10^4^ copies

10^6^ copies

**Supplementary Fig. 3** Mean daily N_2_O emissions measured after conversion October 2008 – October 2009. Measurements of emissions from a standard area over a defined time period were converted to “per g soil per day” by assuming that the top 23 cm soil is active and contains 32x10^8^ g ha^-1^. Values were log-transformed to meet assumptions for ANOVA which indicated significant differences (F_8,72_ = 17.1; *p* < .001), the standard errors of differences of means (s.e.d.) for all groups is shown. Multiple comparisons of means were subjected to a post-hoc Tukey test, those significantly different from the unchanged permanent plot from which they were derived are indicated *.

| **OTU** | **SIMPER** contributed/  culmulative | | **Bare fallow (bf)**  converted to | | | **Arable (ar)**  converted to | | | **Grass (gr)**  converted to | | |
| --- | --- | --- | --- | --- | --- | --- | --- | --- | --- | --- | --- |
|  |  |  | **bf** | **ar** | **gr** | **ar** | **bf** | **gr** | **gr** | **bf** | **ar** |
| Verrucomicrobia  g DA101 | 16.3 | 16.3 | **6.90** | 6.43 | 4.44 | **14.60** | 15.00 | 11.90 | **24.30** | 20.40 | 22.30 |
| Acidobacteria  o iii1-15 | 3.5 | 19.8 | 4.68 | 4.60 | 4.14 | 7.43 | 6.65 | 6.91 | 7.63 | 6.51 | 7.03 |
| α-Proteobacteria  g *Kaistobacter* | 2.6 | 22.3 | **4.14** | 2.82 | 1.53 | **2.79** | 3.52 | 1.65 | **0.67** | 2.08 | 1.20 |
| Acidobacteria  f Koribacteraceae | 2.2 | 24.6 | 1.82 | 2.50 | 3.28 | 0.79 | 0.65 | 0.48 | 0.46 | 0.64 | 0.71 |
| Firmicutes  g *Bacillus* | 1.7 | 26.3 | 2.00 | 2.66 | 2.37 | 0.92 | 1.89 | 1.23 | 0.62 | 1.57 | 1.08 |
| Acidobacteria  g *Ca*.  Koribacter | 1.5 | 27.8 | 1.44 | 1.53 | 1.93 | 0.47 | 0.48 | 0.25 | 0.32 | 0.44 | 0.52 |
| β-Proteobacteria  o SC-I-84 | 1.4 | 29.1 | **2.85** | 1.80 | 1.67 | 1.11 | 0.83 | 0.86 | 0.77 | 0.91 | 1.16 |
| α-Proteobacteria  g *Rhodoplanes* | 1.4 | 30.5 | **1.83** | 2.51 | 2.57 | 3.56 | 2.91 | 3.56 | 3.32 | 3.23 | 3.37 |
| Gemmatimonadetes  f Ellin5301 | 1.4 | 31.9 | **2.43** | 1.14 | 0.78 | 0.68 | 1.13 | 0.37 | 0.19 | 0.64 | 0.29 |
| δ-Proteobacteria  f Syntrophobacteraceae | 1.3 | 33.2 | 0.65 | 0.87 | 0.49 | 1.29 | 1.13 | 1.23 | **1.72** | 1.66 | 2.17 |
| Acidobacteria  o RB41 | 1.3 | 34.4 | 1.36 | 1.22 | 0.29 | 1.14 | 1.00 | 0.90 | 0.45 | 0.57 | 0.49 |
| Actinobacteria  o 0319-7L14 | 1.2 | 35.6 | **0.20** | 0.24 | 0.18 | 0.87 | 0.84 | 1.38 | 1.08 | 1.39 | 1.36 |
| Nitrospirae  f 0319-6A21 | 1.0 | 36.6 | 0.60 | 0.62 | 0.23 | 1.46 | 0.91 | 1.38 | 1.04 | 1.02 | 1.27 |
| Gemmatimonadetes  o N1423WL | 1.0 | 37.6 | **1.27** | 1.08 | 0.74 | **0.51** | 0.62 | 0.30 | **0.02** | 0.13 | 0.05 |
| β-Proteobacteria  o Ellin6067 | 1.0 | 38.5 | **1.31** | 1.14 | 1.42 | 0.49 | 0.47 | 0.51 | 0.43 | 0.25 | 0.27 |
| Acidobacteria  f RB40 | 1.0 | 39.5 | **0.14** | 0.27 | 0.52 | 1.02 | 0.46 | 0.89 | 1.29 | 0.77 | 1.19 |

**Supplementary Table 2**. SIMPER (similarity percentage) analysis of 16S amplicon data at OTU: Phylum (sub-Phylum for Proteobacteria) name is followed by the best identification to level o – order; f – family, g – genus with established genera shown in italics. Values are the mean % OTU for three replicates of each treatment. ANOVA post-hoc comparisons of means significantly different in the permanent treatment are indicated in **bold**; significantly different means within groups compared to the corresponding permanent treatment are underlined*.*

| **OTU** | **SIMPER** contributed/  culmulative | | **Bare fallow** | | **Arable** | | **Grass** | |
| --- | --- | --- | --- | --- | --- | --- | --- | --- |
|  |  |  | **2008** | **2011** | **2008** | **2011** | **2008** | **2011** |
| Verrucomicrobia g DA101 | 9.7 | 9.7 | **7.11** | 6.90 | 12.40 | 14.60 | **13.80** | 24.30 |
| α-Proteobacteria g *Kaistobacter* | 5.7 | 15.4 | **9.88** | 4.14 | **4.87** | 2.79 | **2.00** | 0.67 |
| Acidobacteria f Koribacteraceae | 3.6 | 19.0 | **6.25** | 1.82 | 0.79 | 0.79 | 0.70 | 0.46 |
| Acidobacteria o iii1-15 | 3.5 | 22.6 | **2.03** | 4.68 | 4.55 | 7.43 | 4.89 | 7.63 |
| γ-Proteobacteria g *Pseudomonas* | 3.0 | 25.6 | 2.56 | 0.61 | 4.32 | 0.27 | 1.70 | 0.25 |
| Acidobacteria *Ca.* Koribacter | 2.7 | 28.3 | **4.67** | 1.44 | 0.68 | 0.47 | 0.66 | 0.32 |
| Bacteroidetes g *Flavobacterium* | 2.5 | 30.8 | 0.47 | 0.39 | 0.86 | 0.19 | 3.90 | 0.35 |
| β-Proteobacteria o SC-I-84 | 2.2 | 32.9 | **4.10** | 2.85 | 1.51 | 1.11 | 1.75 | 0.77 |
| Bacteroidetes f Chitinophagaceae | 1.9 | 34.9 | **1.82** | 2.51 | **3.43** | 2.44 | **4.73** | 2.92 |
| δ-Proteobacteria o Myxococcales | 1.7 | 36.6 | **1.09** | 2.93 | **2.30** | 3.14 | **3.67** | 2.96 |
| Firmicutes o Bacillales | 1.6 | 38.2 | **2.82** | 2.00 | 1.09 | 0.92 | 0.60 | 0.62 |
| Acidobacteria o RB41 | 1.6 | 39.8 | 1.43 | 1.36 | 2.03 | 1.14 | 0.82 | 0.45 |
| α-Proteobacteria g *Rhodoplanes* | 1.4 | 41.2 | **1.40** | **1.83** | 2.43 | 3.56 | **2.28** | 3.32 |
| γ-Proteobacteria f Sinobacteraceae | 1.3 | 42.5 | **0.54** | **0.45** | 1.82 | 1.50 | 2.28 | 1.35 |
| Gemmatimonadetes f Ellin5301 | 1.1 | 43.6 | **0.91** | 2.43 | 0.67 | 0.68 | **0.21** | **0.19** |
| Gemmatimonadetes o N1423WL | 1.1 | 44.6 | **1.49** | **1.27** | **0.71** | 0.51 | **0.02** | 0.02 |
| Actinobacteria f Gaiellaceae | 1.0 | 45.6 | 1.35 | 2.64 | 1.62 | 2.39 | 1.35 | 2.06 |
| Acidobacteria o Solibacterales | 1.0 | 46.6 | **1.71** | **1.35** | 0.89 | 0.76 | 0.40 | 0.32 |
| δ-Proteobacteria f Syntrophobacteraceae | 1.0 | 47.6 | **0.49** | **0.65** | 1.22 | 1.29 | **1.67** | **1.72** |
| β-Proteobacteria f Oxalobacteraceae | 1.0 | 48.5 | **1.50** | 0.83 | 0.51 | 0.13 | 0.46 | 0.04 |
| Verrucomicrobia f auto67_4W | 1.0 | 49.5 | **0.05** | **0.05** | 0.94 | 0.66 | **1.29** | 1.00 |

**Supplementary Table 3** SIMPER analysis of 16S amplicon data OTU ranking the groups contributing 1% or more of the differences observed between permanent plots in 2008 and 2011, showing the mean % amplicons in each treatment for each year assigned to each group. Phylum (sub-Phylum for Proteobacteria) name is followed by the best identification to level o – order; f – family, g – genus with established genera shown in italics. ANOVA post-hoc comparisons of means significantly different between the plots in either 2008 and 2011 are indicated in **bold**; significantly different means between permanent treatment at the two sampling times are underlined*.*

| **OTU** | **SIMPER %**  contributed/  culmulative | | **Bare fallow (bf)**  converted to | | | | | **Arable (ar)**  converted to | | | | | | **Grass (gr)**  converted to | | | | | |
| --- | --- | --- | --- | --- | --- | --- | --- | --- | --- | --- | --- | --- | --- | --- | --- | --- | --- | --- | --- |
|  |  |  | **bf** | **ar** | | **gr** | | **ar** | | **bf** | | **gr** | | **gr** | | **bf** | | **ar** | |
| unidentified Ascomycota | 4.3 | 4.3 | 5.23 | | 2.89 | | 5.80 | | 6.32 | | 3.16 | | 6.76 | | 7.34 | | 8.33 | | 5.23 |
| Ascomycota  *Gibberella* | 3.7 | 8.0 | 3.59 | | 6.54 | | 1.83 | | **9.25** | | 5.65 | | 1.64 | | **0.78** | | 5.43 | | 3.59 |
| unidentified Ascomycota | 3.4 | 11.4 | **9.30** | | 7.10 | | 9.40 | | 9.16 | | 11.10 | | 8.80 | | **2.83** | | 11.00 | | 9.30 |
| Basidiomycota c Agaricomycetes | 3.1 | 14.5 | 0.02 | | 0.02 | | <0.01 | | 0.03 | | 0.03 | | 0.02 | | **12.70** | | 0.99 | | 0.02 |
| Ascomycota *Fusarium* | 3.0 | 17.6 | 1.90 | | 1.91 | | 0.63 | | 2.99 | | 6.04 | | 0.90 | | 1.64 | | 8.02 | | 1.90 |
| Basidiomycota c Agaricomycetes | 2.8 | 20.4 | <0.01 | | 0.12 | | 0.26 | | 0.25 | | <0.01 | | 0.13 | | 11.80 | | 0.19 | | <0.01 |
| unidentified Basidiomycota | 2.6 | 23.0 | 4.82 | | 3.02 | | 2.32 | | 3.23 | | 5.51 | | 2.00 | | 4.39 | | 2.21 | | 4.82 |
| Ascomycota *Gibberella* | 2.6 | 25.5 | 3.13 | | 6.34 | | 1.36 | | 5.43 | | 2.63 | | 1.01 | | 0.33 | | 2.23 | | 3.13 |
| Ascomycota *Plectosphaerella* | 2.3 | 27.8 | 2.69 | | 1.29 | | 3.76 | | 0.33 | | 4.94 | | 3.15 | | 0.72 | | 1.99 | | 2.69 |
| Ascomycota *Plectosphaerella* | 1.9 | 29.7 | 1.88 | | 0.42 | | 2.96 | | 0.27 | | 4.95 | | 1.86 | | 0.33 | | 0.78 | | 1.88 |
| Ascomycota *Cladosporium* | 1.8 | 31.6 | 0.83 | | 1.60 | | **4.65** | | 0.94 | | 1.22 | | **4.55** | | 0.39 | | 1.34 | | 0.83 |
| Basidiomycota  o Filobasidiales | 1.7 | 33.3 | 4.57 | | 1.60 | | 2.18 | | 2.50 | | 3.97 | | 2.67 | | **0.31** | | 1.71 | | 4.57 |
| Ascomycota f Mycosphaerellaceae | 1.6 | 34.9 | 0.61 | | 3.18 | | 2.54 | | 1.46 | | 1.47 | | 3.99 | | 0.35 | | 1.34 | | 0.61 |
| Basidiomycota c Tremellomycetes | 1.6 | 36.5 | 3.60 | | 1.83 | | 3.47 | | **4.20** | | 1.64 | | 1.95 | | **1.16** | | 4.09 | | 3.60 |
| Unidentified  Fungus | 1.6 | 38.1 | 2.81 | | 1.64 | | 4.72 | | 3.01 | | 2.08 | | 4.42 | | 1.64 | | 2.20 | | 2.81 |
| unidentified Ascomycota | 1.5 | 39.6 | 2.90 | | 2.93 | | 4.65 | | 5.38 | | 3.16 | | 4.42 | | 1.87 | | 3.33 | | 2.90 |
| Basidiomycota c Agaricomycetes | 1.4 | 41.0 | 0.04 | | 0.01 | | 0.09 | | 0.08 | | <0.01 | | 0.01 | | 5.94 | | 0.04 | | 0.04 |
| Ascomycota o Helotiales | 1.4 | 42.3 | 2.56 | | 3.57 | | 0.51 | | 0.73 | | 0.04 | | 0.05 | | 0.09 | | 0.15 | | 2.56 |
| *Zygomycota f Mortierellaceae | 1.4 | 43.7 | 2.91 | | 1.78 | | 1.79 | | 3.53 | | 1.07 | | 1.55 | | **0.38** | | 1.63 | | 2.91 |
| Zygomycota f Mortierella | 1.3 | 45.0 | 3.58 | | 1.43 | | 0.60 | | 0.71 | | 1.75 | | 0.17 | | 0.01 | | 0.05 | | 3.58 |
| Basidiomycota c Agaricomycetes | 1.3 | 46.3 | <0.01 | | <0.01 | | <0.01 | | <0.01 | | <0.01 | | <0.01 | | **5.26** | | 0.43 | | <0.01 |
| Zygomycota *Mortierella* | 1.2 | 47.6 | 2.76 | | 1.92 | | 0.24 | | 0.78 | | 1.73 | | 0.23 | | 0.13 | | 0.18 | | 2.76 |
| Ascomycota f Nectriaceae | 1.2 | 48.8 | 0.41 | | 0.08 | | 0.48 | | 0.15 | | 1.95 | | 0.47 | | 0.68 | | 3.27 | | 0.41 |
| Basidiomycota  o Agaricales | 1.0 | 49.8 | <0.01 | | <0.01 | | <0.01 | | <0.01 | | <0.01 | | <0.01 | | <0.01 | | <0.01 | | <0.01 |

**Supplementary Table 4** SIMPER analysis of ITS amplicon data at OTU level: Phylum name is followed by the best identification to level o – order; f – family, established genera shown in italics. Values are the mean % OTU for three replicates of each treatment. ANOVA post-hoc comparisons of means significantly different in the permanent treatment are indicated in **bold**; significantly different means within groups compared to the corresponding permanent treatment are underlined*.*
